# Three-Dimensional Maps of All Chromosomes in Human Male Fibroblast Nuclei and Prometaphase Rosettes

**DOI:** 10.1371/journal.pbio.0030157

**Published:** 2005-04-26

**Authors:** Andreas Bolzer, Gregor Kreth, Irina Solovei, Daniela Koehler, Kaan Saracoglu, Christine Fauth, Stefan Müller, Roland Eils, Christoph Cremer, Michael R Speicher, Thomas Cremer

**Affiliations:** **1**Department of Biology II, Anthropology and Human GeneticsLudwig Maximilians University, MunichGermany; **2**Kirchhoff Institute of Physics, University of HeidelbergHeidelbergGermany; **3**Theoretical Bioinformatics, German Cancer Research Center (DKFZ)HeidelbergGermany; **4**Institute of Human Genetics, Technical University MunichGermany; **5**Institute of Human Genetics, GSF National Research Center for Environment and HealthNeuherbergGermany; National Cancer InstituteUnited States of America

## Abstract

Studies of higher-order chromatin arrangements are an essential part of ongoing attempts to explore changes in epigenome structure and their functional implications during development and cell differentiation. However, the extent and cell-type-specificity of three-dimensional (3D) chromosome arrangements has remained controversial. In order to overcome technical limitations of previous studies, we have developed tools that allow the quantitative 3D positional mapping of all chromosomes simultaneously. We present unequivocal evidence for a probabilistic 3D order of prometaphase chromosomes, as well as of chromosome territories (CTs) in nuclei of quiescent (G0) and cycling (early S-phase) human diploid fibroblasts (46, XY). Radial distance measurements showed a probabilistic, highly nonrandom correlation with chromosome size: small chromosomes—independently of their gene density—were distributed significantly closer to the center of the nucleus or prometaphase rosette, while large chromosomes were located closer to the nuclear or rosette rim. This arrangement was independently confirmed in both human fibroblast and amniotic fluid cell nuclei. Notably, these cell types exhibit flat-ellipsoidal cell nuclei, in contrast to the spherical nuclei of lymphocytes and several other human cell types, for which we and others previously demonstrated gene-density-correlated radial 3D CT arrangements. Modeling of 3D CT arrangements suggests that cell-type-specific differences in radial CT arrangements are not solely due to geometrical constraints that result from nuclear shape differences. We also found gene-density-correlated arrangements of higher-order chromatin shared by all human cell types studied so far. Chromatin domains, which are gene-poor, form a layer beneath the nuclear envelope, while gene-dense chromatin is enriched in the nuclear interior. We discuss the possible functional implications of this finding.

## Abstract 2

Through advanced microscopy and labeling techniques these authors can visualize and identify all of the chromosomes in a human nucleus--a key landmark towards understanding how our genome is regulated

## Introduction

Somatic cells within an organism possess genomes that are, with only a few minor exceptions, identical. However, various cell types may possess different epigenomes including the variation of DNA methylation and histone modification patterns. Epigenome variability accounts for cell-type-specific gene expression and silencing patterns in multicellular organisms. The impact of higher-order nuclear architecture on these patterns is not yet known [1]. Studies of higher-order chromatin arrangements in numerous cell types from different species form an indispensable part of a comprehensive approach to understanding epigenome evolution and cell-type-specific variability. Numerous research groups have attempted to map the large-scale organization and distribution of chromatin in cycling and postmitotic cell types (for reviews see [2,3,4,5,6,7,8]). Reliable topological maps, however, for the three-dimensional (3D) and 4D (3D plus spatiotemporal) arrangements of the two haploid chromosome complements in a diploid somatic cell nucleus have been lacking so far. Such 3D and 4D maps would provide the necessary foundation for studying the effect of higher-order chromatin distribution on nuclear functions, and are needed for different cell types at various stages of the cell cycle and at various stages of terminal differentiation. In addition to their importance for epigenome research, these maps should also help to understand karyotype evolution [9,10,11,12] and the formation of chromosomal rearrangements in irradiated or cancer cells [13,14,15,16,17].

In a 2D analysis of human fibroblast prometaphase rosettes, Nagele et al. [18,19] measured distances and angular separations for a number of chromosomes. These authors concluded that the maternal and paternal chromosome sets were separate, and that the heterologous chromosomes in each set showed highly nonrandom distributions. Subsequent studies further emphasized a highly ordered chromosome territory (CT) pattern for the nuclei of polarized human bronchial epithelial cells [20] and for nuclei of quiescent (G0) diploid (46, XY) human fibroblasts in culture [21]. Koss [20] reported that angles between the center of the nucleus and homologous pairs of Chromosome 1, 7, and X CTs were nearly identical in about two-thirds of bronchial epithelial cell nuclei to the angles reported by Nagele et al. for the same chromosome pairs in fibroblast prometaphase rosettes [18]. In contrast, Allison and Nestor [22] found a relatively random array of chromosomes on the mitotic ring of prometaphase and anaphase cells in cultured human diploid fibroblasts, diploid cells from human lung tissue, and human lymphocytes. The causes of these discrepancies have so far remained elusive.

For nuclei of human lymphocytes, phytohemagglutinin-stimulated lymphoblasts, and lymphoblastoid cell lines, several groups have consistently reported a preferential positioning of gene-rich CTs (e.g., Homo sapiens chromosome [HSA] 19) towards the center of the nucleus, and of gene-poor CTs (e.g., HSAs 18 and Y) towards the nuclear periphery [23,24,25,26]. We recently confirmed this gene-density-correlated radial CT positioning for several other normal and malignant human cell types [26]. Bickmore and colleagues [23,27] also reported gene-density-correlated CT arrangements for cycling human fibroblasts. In contrast, Sun et al. [28] and our group [23,24,25,26] provided support for chromosome-size-correlated radial arrangements in quiescent fibroblasts. Although Sun et al. refer to nuclei studied in the G1-phase of the cell cycle, we believe that most of the cells included in their analysis were in a quiescent state (G0), since fibroblasts were grown on coverslips to 90%–95% confluence. Bridger et al. [27] reported that Chromosome 18 CTs were significantly closer to the nuclear periphery in S-phase fibroblasts than in quiescent fibroblasts. These findings suggest that cycling and noncycling fibroblasts differ in higher-order chromatin organization. We tested this hypothesis further in the present study.

To overcome some of the technical limitations of previous studies, and to explore some of their inconsistencies, we employed 3D fluorescence in situ hybridization (FISH) protocols that allowed the differential coloring of all 24 chromosome types (22 autosomes plus X and Y) simultaneously within a population of human male fibroblasts (46, XY) under conditions preserving the 3D nuclear shape and structure to the highest possible degree [29,30]. In addition, we performed a series of two-color 3D FISH experiments in semi-confluent cultures, and determined the radial 3D positions of a subset of CTs (HSAs 1, 17–20, and Y) in quiescent (G0) and cycling (early S-phase) fibroblasts. Our data demonstrate unequivocally that the 3D arrangements of chromosomes in quiescent and cycling human fibroblasts follow probabilistic rules, and suggest that nuclear functions in human fibroblasts do not require a deterministic neighborhood pattern of homologous and heterologous chromosomes. Throughout, when we use the term “probabilistic chromosome order,” we mean an order that cannot be explained simply as a consequence of geometrical constraints that affect the distribution of chromosomes in mitotic rosettes or of CTs in cell nuclei. Constraints may enforce an arrangement of large and small chromosomes or CTs that deviates significantly from the prediction of a random order of points without any functional implications. Our long-term goal is to contribute to the elucidation of the set of rules (most likely a combination of probabilistic and deterministic) that generate cell-type-specific, functionally relevant higher-order chromatin arrangements.

## Results

### Differential Coloring of All 24 Chromosome Types in Nuclei of Human Male Diploid Fibroblasts

**Figure 1:**
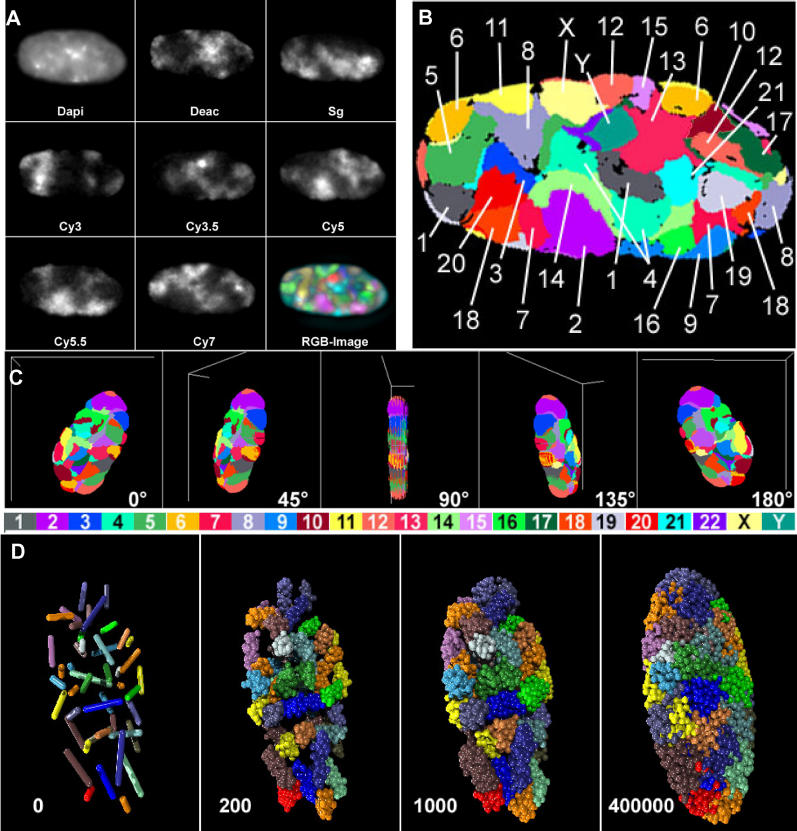
24-Color 3D FISH Representation and Classification of Chromosomes in a Human G0 Fibroblast Nucleus (A) A deconvoluted mid-plane nuclear section recorded by wide-field microscopy in eight channels: one channel for DAPI (DNA counterstain) and seven channels for the following fluorochromes: diethylaminocoumarin (Deac), Spectrum Green (SG), and the cyanine dyes Cy3, Cy3.5, Cy5, Cy5.5, and Cy7. Each channel represents the painting of a CT subset with the respective fluorochrome. The combinatorial labeling scheme is described in Materials and Methods. RGB images of the 24 differently labeled chromosome types (1–22, X, and Y) were produced by superposition of the seven channels (bottom right). (B) False color representation of all CTs visible in this mid-section after classification with the program goldFISH. (C) 3D reconstruction of the complete CT arrangement in the nucleus viewed from different angles. (D) Simulation of a human fibroblast model nucleus according to the SCD model (see Materials and Methods). The first image shows 46 statistically placed rods representing the 46 human chromatids. The next three images simulate the decondensation process and show the resulting CT arrangement obtained after different numbers of Monte Carlo relaxation steps (200, 1,000, and 400,000). This set of figures is taken from Video S1.

Early-passage human fibroblast cultures (46, XY) were grown to confluence and maintained at this stage for several days before being fixed with buffered 4% paraformaldehyde. Under these conditions, the overwhelming majority (>99%) of cells were postmitotic (G0), as demonstrated by a lack of both pKi67 staining and incorporation of thymidine analogs (data not shown). Two 3D multiplex FISH (M-FISH) protocols were used for the differential coloring of all 24 human chromosome types (22 autosomes plus X and Y). The first approach was based on 3D M-FISH with 24 chromosome paint probes. Probes were differentially labeled using a combinatorial labeling scheme with seven different haptens/fluorochromes [31]. DAPI was used to stain nuclear DNA. Light-optical serial sections were separately recorded for each fluorochrome using digital wide-field epifluorescence microscopy (Figure 1). A second approach, called ReFISH [32], achieved differential staining of all 24 human chromosome types in two sequential FISH experiments with triple-labeled probe subsets. Light-optical serial sectioning of the same nuclei with laser confocal microscopy was performed after both the first and the second hybridization. Both approaches provided stringent accuracy for color classification of all CTs, and yielded the same results. Therefore, we combined data from 31 nuclei studied with the first approach and from 23 nuclei studied with the second approach (54 nuclei in total).

Following careful correction for chromatic shifts, and image deconvolution in the case of wide-field microscopy (Figure S1), we performed overlays of the corresponding light-optical sections from all channels with voxel accuracy. CT classification was carried out on these overlays by the computer program goldFISH [33] (Figures 1B and S1C). This program classifies chromosomes by virtue of differences in the combinatorial fluorescent labeling schemes. Figure 1C shows the 3D reconstruction of a nucleus with all CTs viewed from different angles. Although the present experiments were not designed to address the issue of chromatin intermingling from neighboring CTs, it is obvious that goldFISH should have led to numerous misclassifications if there were excessive, widespread intermingling (for further discussion of CT boundaries, see [34]). For each individual CT the classification achieved by goldFISH was confirmed or rejected by careful visual inspection of light-optical sections. Any CT that could not be classified with certainty was omitted from further consideration. We were thus able to identify 2,030 CTs (82%) from a total of 2,484 CTs present in the 54 diploid fibroblast nuclei.

As reference points for all distance and angle measurements reported below, we determined the 3D location of the fluorescence intensity gravity centers (IGCs) of individual painted CTs and the IGC of the nucleus (CN). Unless stated otherwise, when we describe below the position of a CT or prometaphase chromosome (PC) and report distance and angle measurements, we are referring to the 3D position of the CT's or PC's IGC. As a control for the reliability of the CT localizations, we subjected nuclei first studied by 24-color 3D FISH to a sequential five-color FISH experiment with individually labeled paint probes for Chromosomes 1 (Cy5), 3 (Cy3), 10 (FITC), 12 (Cy3.5), and 20 (Cy5.5). We were able to retrieve 11 of the 31 originally studied nuclei and to determine whether 3D positions of CTs first classified in the 24-color 3D FISH experiment could be confirmed after the second hybridization. In 96% of the re-hybridized CTs, the 3D position of the IGC differed by less than 1 μm, the range being between 0.01 and 1.3 μm.

### Size-Correlated Radial CT Positions in Nuclei of Quiescent (G0) Fibroblasts

**Figure 2:**
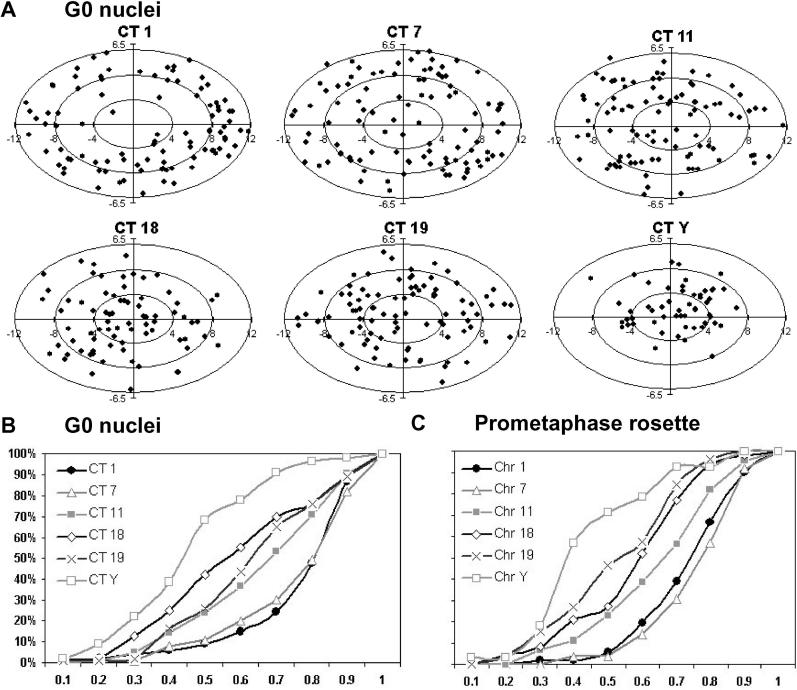
Radial Chromosome Positions Correlate with Chromosome Size in Quiescent Human Fibroblasts (G0) (A) Two-dimensional projections of the IGCs of CTs 1, 7, 11, 18, 19, and Y studied in 54 nuclei are represented by dots. Ellipses represent the 2D shape of a fibroblast nucleus normalized for shape and size and rotated so that the long axis of each nucleus evaluated lies on the abscissa. Projections of IGCs for all CTs are shown in Figure S2. Note that we were not able to distinguish in nuclei between a “north” and “south” pole of the short axis or a “west” and “east” pole of the long axis. Either fibroblast nuclei do not possess such compass polarizations or we lack markers to recognize them. Accordingly, distance comparisons between IGCs located in different quadrants of the ellipse are not meaningful. (B) Cumulative 3D distance graphs of the CT distribution within a normalized nucleus taken from the data (A). The abscissa represents the normalized radial 3D distances of CTs 1, 7, 11, 18, 19, and Y from the center of the nucleus (CN; IGC of the DAPI-stained nucleus) to the IGC of a specific CT. The origin represents the CN, and “1” represents the nuclear periphery. The Ordinate represents the cumulative percentage of normalized 3D CN–CT distances. Cumulative graphs for the entire set of chromosomes are shown in Figure S4. (C) Cumulative 3D distance graphs of PC distribution within a normalized prometaphase rosette. Abscissa and ordinate are as in (B), with PC being the IGC of a prometaphase chromosome and CR the center of the prometaphase rosette. IGC projections and cumulative graphs for all PCs are shown in Figures S3 and S6, respectively.

**Figure 3:**
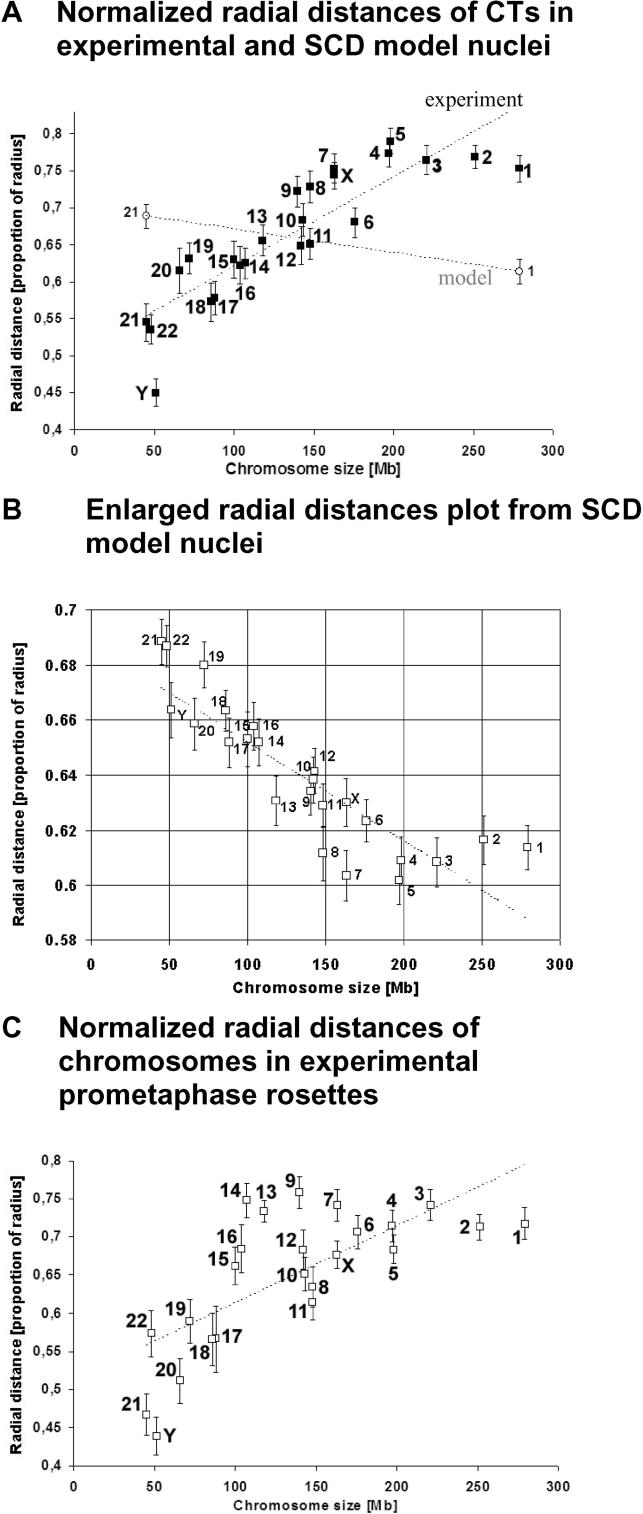
Normalized Radial Chromosome Distances in G0 Fibroblast Nuclei, SCD Model Nuclei, and Prometaphase Rosettes (A) Normalized 3D radial CN–CT distances (filled squares) show a positive correlation with chromosome size (indicated by DNA content): CTs of small chromosomes were preferentially located in the center of the nucleus, whereas CTs of large chromosomes were found more often at the nuclear periphery. Open circles 1 and 21 indicate the endpoints of SCD model data simulating a statistical placement of CTs. (B) SCD model data indicate that geometrical constraints result in a reverse pattern of CT distributions, i.e., modeled small CTs show a significantly higher probability of being localized at the nuclear periphery, while modeled large CTs adopt a more internal localization. (C) In agreement with normalized 3D CN–CT distances, normalized 3D CR–PC distances also show a positive correlation with the DNA content or size of chromosomes: small PCs were preferentially located near the CR, large chromosomes at the rosette periphery.

For every identified CT we measured the 3D radial CN–CT distance (from the CN to the CT's IGC). For a graphic overview of the location of each CT in 2D nuclear projections, the 3D positions of all IGCs obtained for a given CT were normalized and drawn into an ellipse representing the nuclear rim (Figure 2). As representative examples, Figure 2A shows nuclear projections of the normalized 3D IGC locations of CTs of HSAs 1, 7, 11, 18, 19, and Y, while Figure 2B shows cumulative 3D CN–CT graphs for the same CTs. Figures S2 and S4 provide the respective data for the entire chromosome complement. Notably, 3D radial CN–CT distance measurements did not reveal a significant difference between the positions of the gene-poor HSA 18 and the gene-rich HSA 19, although distinctly peripheral and interior locations, respectively, have been found for these two chromosomes in the spherical nuclei of lymphocytes and several other cell types (see Introduction). In summary, our data (Figures 2B, S2, S4, and S7 [left panel]) demonstrate that the territories of all small chromosomes—independent of their gene density—were preferentially found close to the center of the nucleus, while the territories of large chromosomes were preferentially located towards the nuclear rim. Figure 3 displays the positive correlation obtained in quiescent human fibroblasts for the mean normalized radial CN–CT distances and the DNA content of the chromosomes. The broad variability of radial CT positions seen in the set of 54 G0 nuclei indicates that radial CT arrangements in quiescent fibroblasts follow probabilistic, not deterministic, rules.

**Figure 4:**
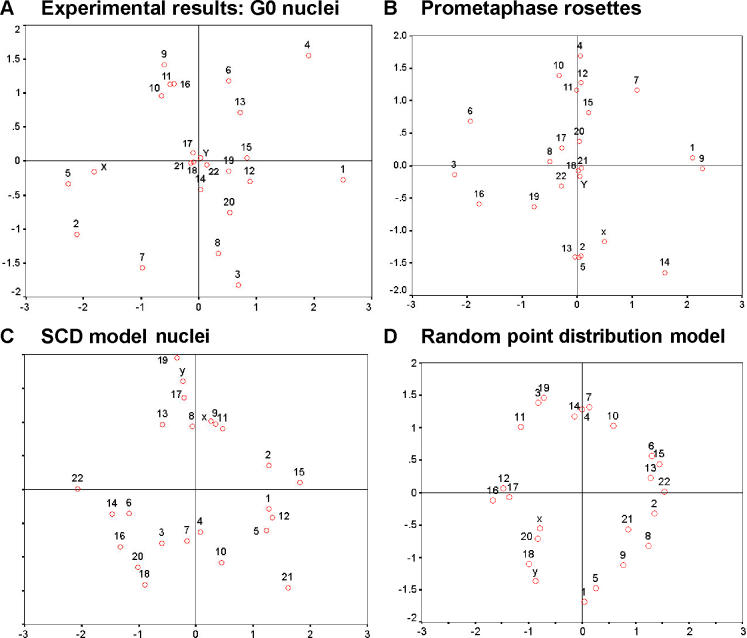
MDS Plots of Experimental and Simulated Heterologous Distances (A) The MDS plot provides a 2D distance map of the mean locations of the IGCs of all heterologous CTs established from 54 G0 nuclei (for further explanations see Materials and Methods). The units (dimension 1 and dimension 2) are arbitrary. (B) MDS plot for all heterologous PCs in 28 prometaphase rosettes. (C) MDS plot for statistically placed CTs in 50 SCD model nuclei. (D) MDS plot for 50 model nuclei with points randomly placed with a random number generator.

To visualize the relative average positions of the IGCs of all heterologous CTs, we generated multidimensional scaling (MDS) plots [35,36] based on the mean of all normalized 3D CT–CT distances (Figure 4). Consistent with the data shown in Figure 3A, we found CTs from small chromosomes preferentially clustering towards the center of the nucleus, while CTs from large chromosomes were preferentially located towards the periphery.

**Figure 5:**
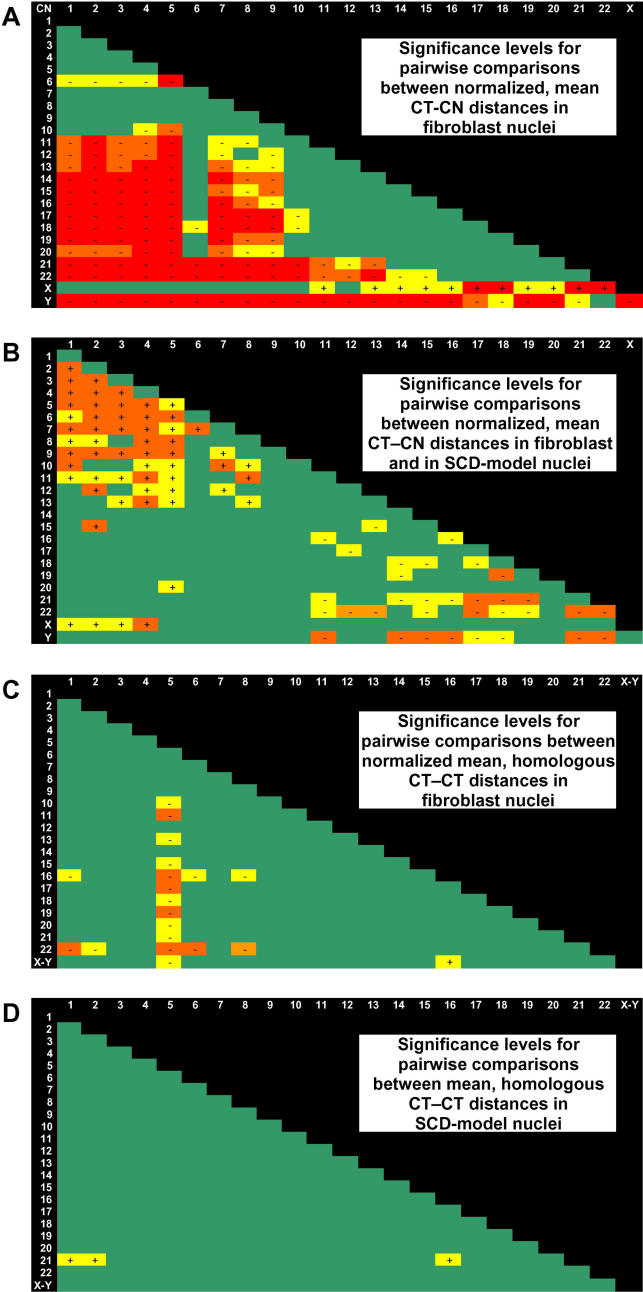
Significance Levels for Pairwise Comparisons of 3D Distance Measurements Performed in 54 G0 Fibroblast Nuclei and in 50 SCD Model Nuclei Significance levels determined by the two-tailed K-S test are indicated by colors. Green, not significant, *p* > 0.01; yellow, *p* < 0.01; orange, *p* < 0.001; red, *p* < 0.0001. A minus (or plus) sign in a colored field indicates that the 3D CN–CT (A and B) or 3D CT–CT distance (C and D) indicated at the left (row) revealed a significantly shorter (or greater) mean radial distance than the CT indicated at the top (column). (A) Comparison of 3D CN–CT distances in G0 fibroblast nuclei. (B) Comparison of 3D CT–CN distances in fibroblast nuclei (vertical row) with 3D CN–CT distances in SCD model nuclei (horizontal row). (C) Comparison of 3D CT–CT distances between homologous chromosomes in G0 fibroblast nuclei. (D) Comparison of 3D CT–CT distances between homologous chromosomes in SCD model nuclei.

The acrocentric chromosomes (13–15, 21, and 22) carry nucleolar organizer regions (NORs) on their short arms, and active NORs are associated with the nucleoli. Since nucleoli are generally located away from the nuclear envelope in the inner nuclear space, we expected that normalized 3D CN–CT distances for all acrocentric chromosomes should be significantly shorter on average than 3D CN–CT distances for the largest chromosomes. Figure 5 confirms this expectation in the sample of 54 3D evaluated nuclei, emphasizing the sensitivity of the IGC approach. We also found a highly significant difference (*p* < 0.001 to *p* < 0.0001; two-tailed Kolmogorov-Smirnov [K-S] test) between the mean radial CT positions of the five largest chromosomes (HSAs 1–5) and those of smaller chromosomes (HSAs 11–22 and Y). The radial positioning of the X territory did not differ significantly from the distribution of the five largest CTs.

### Size-Correlated Radial Chromosome Positions in Human Fibroblast Prometaphase Rosettes

Growing fibroblast cultures (46, XY) were subjected to 24-color 3D FISH. The shape of prometaphase rosettes identified in these cultures resembled an intermediate between a flat cylinder and a hemisphere. Figure S5 shows a typical 3D-reconstructed rosette, as well as light-optical sections before and after deconvolution and after classification of PCs. In a sample of 28 rosettes with a total of 1,288 PCs, we determined the IGC of the prometaphase rosette (CR) and were able to classify 990 PCs (77%) by goldFISH and visual inspection. Consistent with the findings described above for CT arrangements in quiescent fibroblast nuclei, we observed a size-correlated radial PC distribution, i.e., IGCs of small PCs were typically clustered closer to the center of the rosette, while IGCs of large PCs were preferentially located towards the rim (see Figures 2C, 3C, 4B, S3, S5, S6, and S7 [right panel]).

### Voxel-Based Comparison of Radial CT Positions in Nuclei from Quiescent (G0) and Cycling Human Fibroblasts

**Figure 6:**
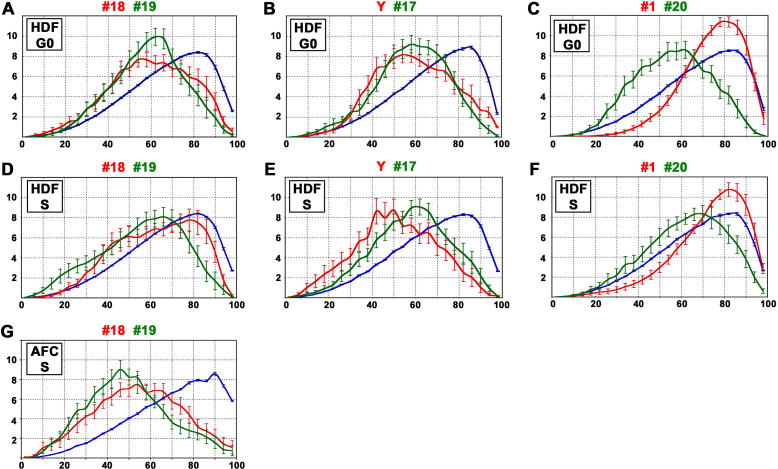
Comparison of Radial CT Positions in Quiescent and Proliferating Cell Nuclei The abscissa shows the radial 3D distribution of CT-specific and whole-DNA-specific voxels in 25 concentric nuclear shells. The origin represents the center of the nucleus, and “100” represents the nuclear border. The ordinate indicates the mean frequency of the intensity-weighted voxels above threshold in each shell (in percent). Example images are shown in Figure S8. Bars correspond to standard errors of the mean. (A–F) A semi-confluent human diploid fibroblast (HDF) culture containing both quiescent cells (G0) and proliferating cells was used for two-color 3D FISH with different pairs of chromosome paint probes. In each experiment light-optical serial sections were recorded from 20 G0 nuclei (A–C) and 20 nuclei at early S-phase (D–F). All voxels attributed to a segmented CT were used to describe its radial position (red and green curves). Blue curves show the voxel-based radial distribution of whole DNA stained with TOPRO-3. Comparison of (A and D) gene-poor HSA 18 (red curve) and gene-rich HSA 19 (green curve), (B and E) gene-poor HSA Y (red curve) and gene-rich HSA 17 (green curve), and (C and F) large HSA 1 (red curve) and small HSA 20 (green curve). Irrespective of their gene content the small HSAs 17, 18, 19, 20, and Y were all located significantly closer towards the nuclear interior compared to the distribution of the whole nuclear DNA (*p* < 0.05, two-tailed K-S test). The difference between the radial location of pair HSA 18/19 CTs both in quiescent and cycling cell nuclei, however, was not significant. For the pair HSA 17/Y, no significant difference was obtained between G0 and S-phase nuclei (*p* > 0.05, Students *t*-test [*t-*test]). In contrast, HSA 1 was significantly shifted towards the nuclear border compared to HSA 20 (*p* < 0.001, *t*-test), although HSAs 1 and 20 have a similar overall gene density. (G) Comparison of HSA 18 (red curve) and HSA 19 (green curve) in nuclei of proliferating amniotic fluid cells during S-phase (*n* = 18). Although there is a slight excess of CT 19 voxels towards the nuclear interior compared to CT 18 voxels, the two curves are not significantly different (*p* > 0.05; *t*-test).

For a direct comparison of the radial CT positioning of gene-poor and gene-rich chromosomes, we performed two-color 3D FISH experiments and painted CTs of HSAs 18/19 and HSA Y/17 in nuclei of quiescent and cycling fibroblasts (Figures 6 and S8). Semi-confluent cell cultures were fixed for this purpose at a density that yielded large fractions of both quiescent and cycling cells. Cultures were pulse-labeled with BrdU immediately before 3D fixation. Cells showing a BrdU labeling pattern typical of early S-phase [37] were chosen for 3D evaluation of cycling cells. Quiescent cells (G0) identified in the same culture lacked pKi67 and BrdU staining (data not shown). We recorded light-optical serial sections from 20 G0 nuclei and 20 S-phase nuclei. Since the use of IGCs for the location of individual CTs has obvious limitations, we used a voxel-based evaluation. After thresholding, each voxel attributed to a given CT was located in one of 25 concentric 3D nuclear shells. To define these shells, the radii in the *x, y,* and *z* directions were subdivided into 25 nuclear segments of equal thickness. The innermost shell comprised the smallest volume, and the outermost shell the largest volume. Figure 6A and 6D show the results for HSA 18 CTs (red) and HSA 19 CTs (green). Figure 6B and 6E show the respective results for the Y territory (red) and HSA 17 CTs (green). In each experiment the normalized CT-specific radial distribution curves were compared with the curve obtained for the TOPRO-3-stained total nuclear DNA (blue). The latter curve was taken as a rough approximation for a uniform radial DNA distribution (neglecting differences in the radial distribution of heterochromatin and euchromatin). This comparison revealed for both quiescent and cycling cells that Chromosome 17, 18, 19, and Y CTs, in spite of the pronounced differences in gene density, were all located significantly closer to the nuclear center than expected in the case of a uniform radial distribution (*p* < 0.05; two-tailed K-S test). In both quiescent and cycling fibroblast nuclei the voxel distribution of the gene-poor HSA 18 CTs was slightly shifted towards the 3D nuclear border compared to the gene-rich HSA 19 CTs (Figure 6A and 6D). This shift, however, was nonsignificant for both quiescent and cycling fibroblasts (*p* > 0.05; Mann-Whitney *U*-test [*U*-test]). In contrast, the gene-poor Y territory was slightly more shifted towards the nuclear interior than the gene-rich HSA 17 CTs (Figure 6B and 6E). This shift was significant for cycling fibroblasts (*p* < 0.05; *U*-test), but not for quiescent fibroblasts. We also compared the radial voxel distributions of the CTs of HSAs 1 and 20 as an example of a large and a small chromosome having similar average gene densities (Figure 6C and 6F). In agreement with the hypothesis of a size-correlated radial arrangement, CTs of HSA 20 were significantly shifted towards the nuclear interior compared to the DNA counterstain and to the CTs of HSA 1 (*p* < 0.001; *U*-test).

A novel approach (J. von Hase and C. Cremer, unpublished data) allowed us to measure the nearest 3D distance of each voxel attributed to HSA 18 CTs and HSA 19 CTs, respectively, from the segmented 3D nuclear periphery. We found that a fraction of the voxels attributed to HSA 18 CTs was located closer to the top and bottom part of the nuclear envelope than voxels attributed to HSA 19 CTs. The difference between the two voxel-based distance distributions, however, was only significant in cycling fibroblasts (*p* < 0.05; K-S test).

### Comparison of Higher-Order Chromatin Arrangements in Nuclei of Human Fibroblasts, Amniotic Fluid Cells, and Lymphocytes

All previously studied cell types that showed gene-density-correlated radial CT arrangements had a more spherical nuclear shape [26]. This prompted the question of whether other cell types, in addition to fibroblasts, with flat-ellipsoidal nuclei would also reveal a chromosome-size-related radial CT arrangement. To answer this question, we performed two-color 3D FISH experiments with HSA 18 and HSA 19 paint probes in cultured human amniotic fluid cells in the same way as described above (Figure 6G). Such cells have a fibroblast-like phenotype with a flat-ellipsoidal nucleus, but they are typically derived from epithelial cells of the urinary tract [38]. Consistent with the fibroblast data described above, we observed that HSA 18 and 19 CTs were similarly distributed (*p* > 0.05; *U*-test), but located significantly closer to the nuclear center than expected in the case of a uniform radial distribution (*p* < 0.01; two-tailed K-S test).

**Figure 7:**
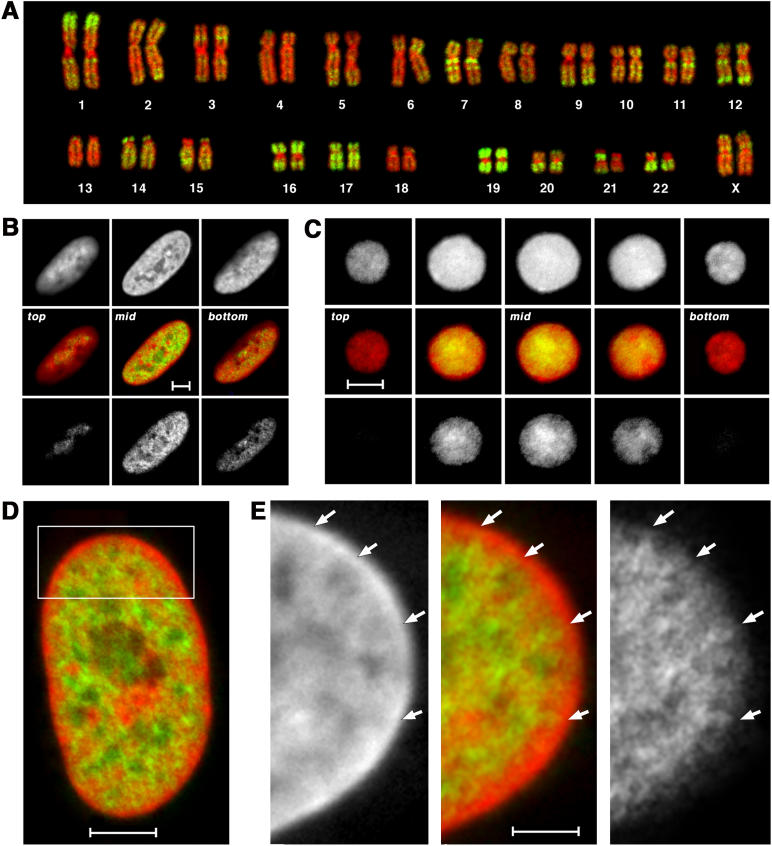
Localization of Alu Sequences in Nuclei of Fibroblasts and Lymphocytes (A) Karyotype from a female human lymphocyte (46, XX). Chromosomes were hybridized with a probe for Alu sequences (green) and counterstained with TOPRO-3 (red). Alu sequences were used as a marker for chromosomes and chromosome bands rich in genes. (B and C) Confocal serial sections were obtained from a human G0 fibroblast nucleus (B) and a G0 lymphocyte nucleus from peripheral blood (C) after 3D FISH with the Alu probe (green) and TOPRO-3 counterstaining (red). As examples, sections made at the top, middle, and bottom of the nuclei (separated by about 1 μm) are shown from left to right. Scale bars, 5 μm. (D) Enlarged confocal mid-section through the human G0 fibroblast nucleus. Scale bar, 5 μm. (E) Enlargement of the boxed sector in (D). The color image in the middle reflects the merged images left (TOPRO-3 counterstaining, red) and right (Alu staining, green). Arrows indicate chromatin rich in Alu sequences expanding into the TOPRO-3-stained, Alu-poor nuclear rim. Scale bar, 2 μm.

For a direct comparison of the 3D distribution of gene-rich and gene-poor chromatin in nuclei of human fibroblasts and lymphocytes from peripheral blood, we studied the 3D nuclear distribution of the repetitive DNA family of interspersed Alu sequences. The density of Alu sequences corresponds to GC-richness and density of housekeeping genes along human chromosomes [39,40]. Accordingly, HSA 18 has a low and HSA 19 a high content of these sequences (Figure 7A). Three-dimensional FISH experiments with a consensus Alu probe demonstrated for both cell types a concentration of Alu-rich chromatin in the nuclear interior, while Alu-poor chromatin formed a shell attached to the nuclear envelope (Figure 7B–7D). We conclude from this experiment that fibroblast and lymphocyte nuclei—in spite of profound differences in their shapes and radial CT arrangements—share this aspect of a gene-density-correlated, nonrandom, higher-order chromatin arrangement. Notably, expansions from the more interior Alu-rich chromatin into the Alu-poor peripheral shell were seen on closer inspection (Figure 7E), indicating complex spatial interactions of chromatin regions with high and low gene density. Our findings support the hypothesis that HSA 18 CTs in fibroblast nuclei are more intensely connected to the top or bottom part of the nuclear envelope than HSA 19 CTs are.

### Chromosome Proximity Patterns in Human Fibroblast Nuclei

Notwithstanding the gene-density-correlated features of higher-order chromatin arrangements described above, both IGC- and voxel-based evaluation procedures consistently demonstrated that radial CT arrangements in nuclei of quiescent and cycling fibroblasts correlate with chromosome size. In an attempt to quantify patterns of proximity of homologous and heterologous chromosomes, we measured 3D CT–CT and 3D PC–PC distances, as well as 3D CT–CN–CT and 3D PC–CR–PC angles within the nuclear space. To assess the possibilities and limitations of such measurements, let us imagine that we could perform “orbital” walks around the nucleus in a series of concentric 3D shells (like planets orbiting around the sun). A priori the evidence for a nonrandom radial CT order is compatible with both random and nonrandom “orbital” arrangements of CTs. Combined 3D CT–CT and 3D CT–CN–CT (or 3D PC–PC and 3D PC–CR–PC) measurements provide an opportunity to discriminate between the two possibilities. If size-correlated radial arrangements were the only principle of an ordered CT or PC positioning in human fibroblasts, we would expect a broad range of angles from very small ones to 180°, independent of the preferred radial position for any pair of CTs/PCs. In contrast, consistently small or large angles for a given chromosome pair would strongly support the notion of nonrandom associations or separations of the two chromosomes.

#### Proximity patterns of homologous chromosomes

**Figure 8:**
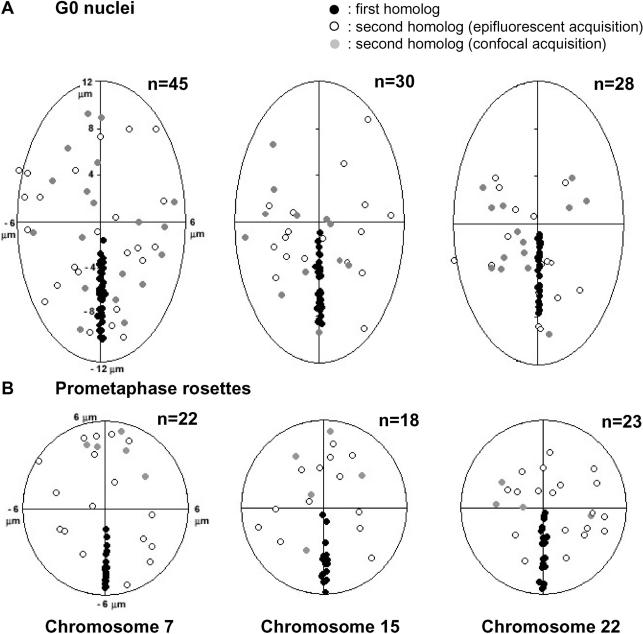
Relative Spatial Distributions of Homologous Chromosomes of HSAs 7, 15, and 22 Schematic outlines of fibroblast nuclei (as ellipses, A) and prometaphase rosettes (as circles, B) with normalized size and shape. The IGC of one randomly selected homolog was placed along the negative long axis. The IGC of the other homolog was marked at the corresponding nuclear position. Gray dots represent data obtained by confocal microscopy, open circles by wide-field microscopy. Angle measurements for all pairs of homologous chromosomes are presented in Figure S9 for G0 nuclei and in Figure S10 for rosettes.

A broad range of normalized 3D CT–CT and 3D PC–PC distances was observed between all homologous chromosome pairs in the 54 G0 fibroblast nuclei and 28 prometaphase rosettes (data not shown). Consistent with this variability 3D CT–CN–CT angles (Figure S9) and 3D PC–CR–PC angles (Figure S10) ranged from very small to large angles near 180°. Figure 8 illustrates the broad angle distributions obtained for homologous chromosome pairs 7, 15, and 22 in both G0 nuclei and prometaphase rosettes. Figure 5C shows the significance levels obtained in G0 fibroblast nuclei for all possible comparisons of normalized distances between two different pairs of homologous CTs. From a total of 276 possible comparisons, 19 yielded a significant difference; 12 of these suggested that the two CTs of HSA 5 were located significantly more distant from each other than the homologous CTs of HSAs 10, 11, 13, and 15–22, as well as the CTs of the two gonosomes, X and Y. Further studies will tell us whether the preferential involvement of HSA 5 is a true finding or a statistical artifact occasionally expected when large numbers of comparisons are statistically tested. In conclusion, these data indicate highly variable proximity patterns for homologous chromosomes in human fibroblast nuclei and prometaphase rosettes. However, we cannot firmly exclude at present the possibility that some homologous chromosome pairs occupy nonrandom “orbital” positions with regard to each other.

#### Proximity patterns of heterologous chromosomes

We performed 3D distance measurements between all possible combinations (*n* = 42,988) of heterologous CTs/PCs. These measurements suggested a certain degree of nonrandom proximity: 7,651 (18%) pairwise 3D CT–CT distance comparisons in G0 nuclei and 3,657 pairwise 3D PC–PC distance comparisons (8.5%) in prometaphase rosettes were statistically significant (*p* < 0.01; two-tailed K-S test). The observed percentages of nonrandom proximity may be explained mostly as a consequence of the nonrandom radial CT/PC arrangements described above, but may also to some degree reflect nonrandom orbital arrangements of heterologous CTs/PCs.

Consistent with the finding that 3D CN–CT distances for acrocentric chromosomes were significantly shorter than 3D CN–CT distances for the largest chromosomes (HSA 1–5; see above), we also found a significantly smaller mean 3D CT–CT distance for acrocentric chromosomes than for the five largest chromosomes (Table 1). The mean 3D CT–CT distance for the five largest chromosomes was also significantly larger than the mean distance for the other small metacentric/submetacentric autosomes (HSAs 16–20) that do not carry NORs. In contrast, the mean 3D CT–CT distances for acrocentric chromosomes were not significantly different from the mean distances for these other small chromosomes.

**Table 1:**
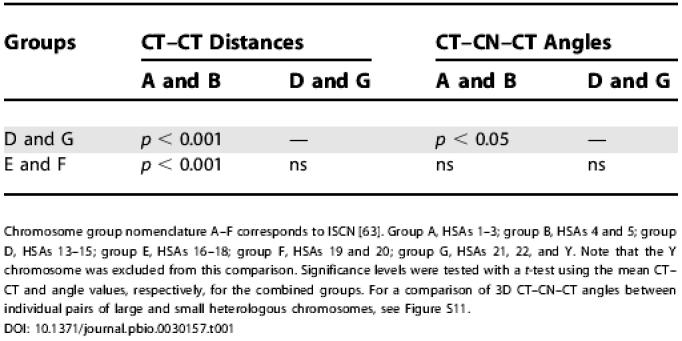
Comparison of Mean 3D CT–CT Distances and Mean 3D CT–CN–CT Angles between Groups of Large (HSAs 1–5) and Small (HSAs 13–22) Chromosomes

Chromosome group nomenclature A–F corresponds to ISCN [63]. Group A, HSAs 1–3; group B, HSAs 4 and 5; group D, HSAs 13–15; group E, HSAs 16–18; group F, HSAs 19 and 20; group G, HSAs 21, 22, and Y. Note that the Y chromosome was excluded from this comparison. Significance levels were tested with a *t*-test using the mean CT–CT and angle values, respectively, for the combined groups. For a comparison of 3D CT–CN–CT angles between individual pairs of large and small heterologous chromosomes, see Figure S11

The mean 3D CT–CN–CT angle obtained for the five largest chromosomes was not significantly different from the mean angle for the small metacentric and submetacentric chromosomes, while a modestly significant difference (*p* < 0.05) was noted in mean angle between the latter and the acrocentric chromosomes (Table 1). Table 2 presents the mean angles with standard deviations and the angle range measured for all possible pairwise combinations of the largest chromosomes (HSAs 1–5), the acrocentric chromosomes (HSAs 13–15, 21 and 22), and the other small chromosomes (HSAs 16–20). For all comparisons we noted a large angle range from a few degrees to more than 170 degrees. Figure S11 presents a comparison of CT–CN–CT angle distributions between different pairs of heterologous CTs. With few exceptions these comparisons did not reveal significant differences.

**Table 2:**
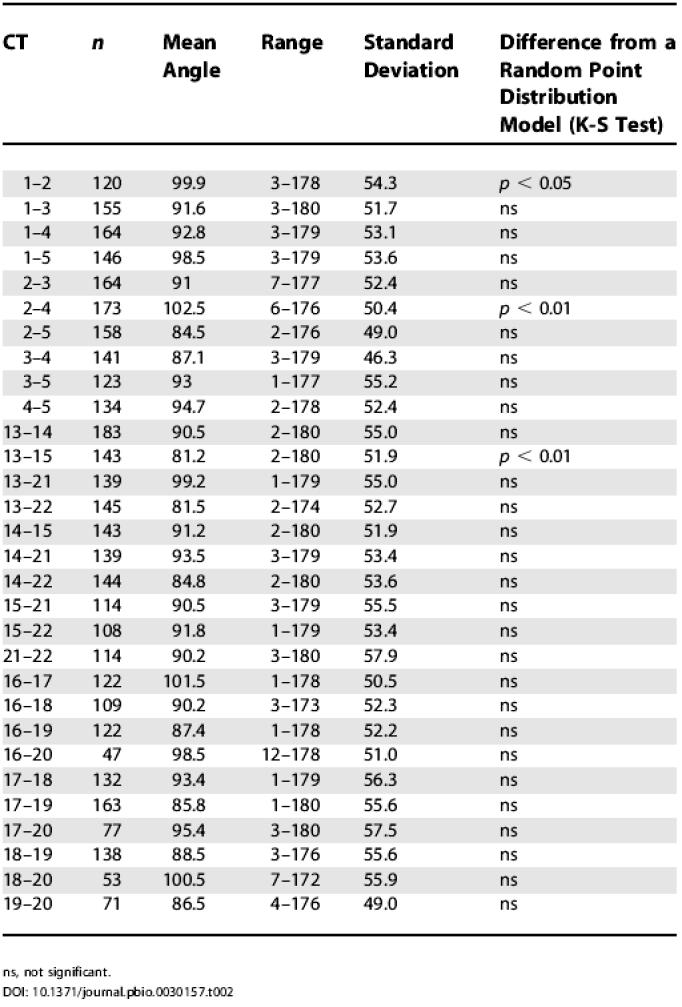
Angular Separation of the Largest and Smallest Heterologous CTs in G0 Nuclei

ns, not significant

While the findings described above support the possibility of nonrandom proximity between chromosomes in fibroblast nuclei, the observed cell-to-cell variability emphasizes the probabilistic nature of these patterns. This large variability of proximity is further emphasized in Figure S12 for CTs of HSAs 7 and 8. It shows projections of confocal image stacks from a series of 50 randomly chosen G0 fibroblast nuclei after two-color 3D FISH with the respective paint probes. Quantitative evaluation of this experiment (Figure S13) confirmed that the angular distribution for these CTs did not significantly differ from that calculated for points randomly placed in an ellipsoid. In conclusion, our results do not support the existence of a highly ordered orbital arrangement of the paternal and maternal chromosome sets.

### Comparison of CT Positions in Fibroblast Nuclei (G0) with Modeled CT Positions

As a first and very simple attempt to create a model for the random distribution of the IGCs from all CTs present in a diploid human fibroblast nucleus, we placed 46 points randomly in ellipsoids with similar shapes, and performed distance and angle measurements between the points and the centers of the ellipsoids (random point distribution model). Twenty-seven out of 30 comparisons of heterologous CT–CN–CT angle distributions measured in fibroblast nuclei did not reveal a significant difference from the random point distribution model (Table 2). The three exceptions were the 3D CT–CN–CT angles between CTs of HSAs 1 and 2 (*p* < 0.05), CTs of HSAs 2 and 4 (*p* < 0.01), and CTs of HSAs 13 and 15 (*p* < 0.01). In prometaphase rosettes (see Figure S10), none of the angle distributions for homologous chromosome pairs differed significantly from the distributions obtained for randomly distributed points.

The random point distribution model does not take into account geometrical constraints, which likely affect the distribution of small and large CTs within the limited nuclear space. Thus, a significant difference between model and experimental results could simply result from geometrical constraints imposed on the distribution of CTs with various sizes. To determine the possible influence of geometrical constraints, we used the spherical 1-Mbp chromatin domain (SCD) model [41], which assumes that CTs are built up from 1-Mbp domains, and that the relative fraction of the nuclear volume occupied by each CT is directly proportional to the number of these domains that constitute a chromosome. Chromosomes of male diploid fibroblasts (46, XY) in telophase/early G1 were modeled as rods with lengths reflecting their relative DNA content, since estimates of CT volumes supported a rough positive correlation with DNA content (data not shown). These rods were statistically placed with random orientations into an ellipsoid with the *x, y,* and *z* diameters corresponding to the average main diameters of the fibroblast nuclei. Using Monte Carlo calculations, we allowed the model chromosomes in these starting configurations to “decondense” to CTs in early G1 nuclei (see Figure 1D; Video S1). Since any modifications of the starting arrangements of rods in a model nucleus may influence the final CT distribution profoundly, this approach should be considered only as an attempt to model the arrangements of all CTs in fibroblast model nuclei with ellipsoidal shape. Note that it was not our goal here to model the real anaphase/telophase chromosome distribution or the chromatin movements involved in the formation of G1 nuclei. Instead, we wished to study whether geometrical constraints acting during the process of decondensation strongly influence the final positions of decondensed CTs compared to the positions of statistically placed condensed chromosomes.

In a set of 50 SCD model nuclei with fully decondensed model CTs, we determined the 3D centers of gravity for the 46 simulated CTs, and performed distance and angle measurements. In contrast to fibroblast nuclei and prometaphase rosettes (see Figure 3A and 3C), 3D radial distances measured in these 50 SCD model nuclei revealed an inverse correlation of radial positioning and chromosome size (see Figure 3B). Large modeled CTs were preferentially located in the interior and small CTs towards the rim of SCD model nuclei. Figure 5B shows the significance levels obtained for the pairwise comparison of 3D CN–CT distances in SCD model nuclei with the corresponding 3D CT–CN distances in fibroblast nuclei. Consistent with this inverse correlation the mean 3D CN–CT (radial) distances measured for the largest chromosomes in real fibroblast nuclei were significantly greater than the mean radial distances for their modeled counterparts in SCD model nuclei. For the smallest chromosomes, we found numerous instances where the mean radial distances measured in fibroblast nuclei were significantly smaller than the SCD model distances. These results suggest that the size-correlated radial CT arrangements observed in fibroblast nuclei cannot be explained simply as a consequence of geometrical constraints affecting the radial positions of small and large CTs.

From a total of 253 possible pairwise comparisons of 3D CT–CT distances between homologous CT pairs in SCD model nuclei, only three (1.2%) were significant (*p* < 0.01; see Figure 5D). The significantly greater 3D distance between modeled CT 21 pairs compared to the 3D distances between modeled CT 1 and CT 2 homologs is in line with the more peripheral location of small versus large modeled CTs. Pairwise comparisons between heterologous 3D CT–CT distances in SCD model nuclei yielded 837 (2%) significant (*p* < 0.01) differences out of 42,988 pairwise distance comparisons. These small percentages emphasize our effort to model a random neighborhood of CTs except for the influence of different geometrical constraints affecting the positions of large versus small CTs. Pairwise comparisons of 3D distances measured in 50 random point distribution model nuclei yielded only 45 out of 42,988 significant differences at a significance level *p* < 0.01, a fraction expected by chance. This comparison emphasizes that geometrical constraints affected the distribution of CTs in SCD model nuclei. The comparison of angle distributions obtained for the 22 homologous autosome pairs and the XY pair in the 50 SCD model nuclei with the respective angle distributions described above for G0 fibroblast nuclei showed no significant differences except for Chromosomes 4 (*p* < 0.01), 7 (*p* < 0.05), 10 (*p* < 0.05), 20 (*p* < 0.01), and 22 (*p* < 0.05) (see Figure S9). In G0 fibroblast nuclei the number of significant differences (*p* < 0.01) was considerably larger for heterologous distance pairs (18%; 7,651/42,988) than for homologous distance pairs (7.5%; 19/253). In conclusion, the considerably higher percentage of nonrandom distance comparisons in G0 fibroblast nuclei (see above) than in SCD model nuclei supports the hypothesis that a certain degree of nonrandom CT proximity exists that is not expected if proximity were simply the result of geometrical constraints imposed on crowded small and large objects (CTs) within a limited volume with special shape (the nucleus). Other mechanisms must be invoked to explain the observed chromosome distributions.

## Discussion

### Probabilistic Chromosome Order in Human Fibroblasts

Mapping the positions of all chromosomes simultaneously in a single nucleus provides a major step forward towards the goal of elucidating general and cell-type-specific rules that govern chromosome positioning in cycling and postmitotic cells. Reviewing the evidence for an “inherently imperfect and probabilistic nature” of CT positioning, Parada et al. [42] came to the following conclusion: “Chromosome positioning patterns are statistical representations of chromosome positions but do not provide information about the precise coordinates of a given chromosome in a given nucleus. It is important to realize that, although significant non-random chromosome positions can be described, they contain a significant degree of uncertainty and merely indicate a preferred, probabilistic position of a given chromosome in the cell nucleus.” This view is corroborated by the present study and cannot be reconciled with previous reports suggesting a precise pattern of chromosomal neighborhood in human fibroblasts [18,19]. Conclusive evidence for preferred, probabilistic rather than absolute CT positioning patterns does not exclude the possibility that higher-order chromatin architecture at large follows determininistic rules to some extent. The observation that a shell of gene-poor chromatin domains is located beneath the nuclear envelope of all cell types studied so far indicates such a deterministic feature.

NOR-bearing CTs placed around nucleoli provide a telling example of how probabilistic and deterministic rules may act together to bring about this well known nuclear topography. The probabilistic combination of NORs from several acrocentric chromosomes gives rise to the formation of the several nucleoli typically found in human fibroblast nuclei. This combination is subject to changes from cell cycle to cell cycle. Consistent with this conclusion we found a large variability of 3D CT–CT distances and CT–CN–CT angles for pairs of homologous and heterologous acrocentric chromosomes. Notwithstanding this variability, distances between CTs of acrocentric chromosomes were significantly smaller than between CTs of the five largest chromosomes, reflecting frequent spatial association of acrocentric chromosomes with the more internally located nucleoli as compared to the high probability of large chromosomes to be located close to the nuclear rim. A probabilistic order of acrocentric CTs as a general feature of human cells is consistent with a quantitative analysis of interchanges between all possible heterologous pairs of chromosomes in human lymphoblast nuclei after damage by sparsely ionizing radiation in vitro [13]. A deterministic feature of this order, however, is introduced by the fact that the formation of a nucleolus at a given nuclear site requires the presence of at least one NOR-bearing chromosome.

Recently, Misteli and coworkers [17,43] determined the nearest neighbors of CTs in cell types of different tissues and found statistically significant cell-type-specific proximity patterns for CT clusters, as well as a correlation between tissue-specific spatial proximity patterns and tissue-specific translocations during tumor development [6]. Considering significant differences between the probabilistic arrangements of CTs/PCs observed in fibroblast nuclei and prometaphase rosettes and the arrangements predicted by random point and SCD model distributions, we must take into account the limited statistical power of the sample sizes evaluated in the present experiments. Repeated experiments with larger sample sizes are necessary to distinguish biologically significant probabilistic proximity patterns unequivocally from potential statistical artifacts.

Since we were not able to distinguish paternal from maternal homologs, the question of a separation of the parental genomes could not be settled by the present experiments. Although the wide range of angles which we measured for each homologous or heterologous chromosome pair does not necessarily exclude the possibility of a spatial separation of the parental genomes, this range clearly argues against a consistent separation of homologous PCs at opposite sites of the rosette [18,19].

### Features of CT Arrangements Correlated with Chromosome Size and Gene Density

In agreement with the study of Sun et al. [28], we found that small chromosomes (independent of gene density) were preferentially located towards the center of fibroblast nuclei, while large chromosomes were preferentially positioned towards the nuclear rim. Evidence for a chromosome-size-correlated radial CT arrangement was also found in nuclei of quiescent and cycling human amniotic fluid cell nuclei ([25,44,45]; present study), as well as in nuclei of cultured orangutan fibroblasts (M. Neusser, T. Cremer, and S. Müller, unpublished data).

The finding of chromosome-size-correlated radial CT arrangements in two cell types with flat-ellipsoidal nuclei contrasts with the gene-density-correlated radial CT arrangements consistently reported for human and other primate cell nuclei with approximately spherical nuclear shapes. There is an apparent discrepancy of our data with reports of the Bickmore group, who described a gene-density-correlated CT arrangement in nuclei of cycling human fibroblasts [24]. This group also reported a central shift of HSA 18 CT positions when fibroblasts became quiescent [27]. In agreement with this finding we also observed HSA 18 CTs somewhat closer to the nuclear periphery in cycling than in quiescent fibroblasts (see Figure 6C). It is not clear at present why the observed differences were much more pronounced in the analysis performed by Bridger et al. [27]. We noted one obvious difference between Bridger et al.'s study and our study: while Bridger et al. studied quiescent fibroblasts in serum-starved cultures, we compared quiescent and cycling cells that coexisted in the same semi-confluent culture supplemented with 10% fetal calf serum (FCS). Serum-starved fibroblasts exhibit extremely flat nuclei compared to cells grown in the presence of FCS (unpublished data). It seems therefore possible that differences in nuclear shape and/or other unknown factors affected the distribution of CTs detected in G0 nuclei from serum-starved fibroblasts compared to G0 nuclei from cells grown in FCS-supplemented medium.

A more complex picture has emerged from our study, namely that both size- and gene-density-correlated features of higher-order chromatin arrangements coexist in human fibroblast nuclei. Chromosome-size-correlated radial CT arrangements are most obvious in 2D nuclear projections (see Figures 2 and S2). In contrast, measurements of HSA 18 and HSA 19 CT positions along the optical axis suggest a location of the gene-poor HSA 18 closer to the top or bottom part of the nuclear envelope than for HSA 19. This hypothesis is supported by our observation that a layer of Alu- and gene-poor chromatin coats the interior side of the nuclear envelope, whereas chromatin rich in Alu sequences and genes was preferentially found in the interior of the nucleus. Considering the fact that the resolution along the optical axis is lower than the lateral resolution, these findings do, of course, not exclude direct contacts of HSA 19 chromatin with the top or bottom part of the nuclear envelope. A detailed comparison of similarities and differences of higher-order chromatin arrangements in nuclei from different cell types is made difficult by the possibility that additional levels of complexity may exist, which were not considered so far. Note, for example, that invaginations of the nuclear membrane may yield a direct contact of chromatin domains or nucleoli apparently detected in the nuclear interior with the nuclear envelope.

### Interdependence between Nuclear Shape and CT Arrangements

The presently available data indicate that CT arrangements differ between cell types with flat-ellipsoidal nuclei, such as human fibroblasts and amniotic fluid cells, and cell types containing nuclei with a more roundish shape [46]. Whether these cell-type-specific differences are generally correlated with or even caused by differences in nuclear shape remains an open question. Does a nuclear shape transformation suffice to change size-correlated CT arrangements observed in flat-ellipsoidal nuclei into gene-density-correlated CT arrangements observed in roundish nuclei, and vice versa? For example, let us consider a population of flat-ellipsoidal nuclei, where the CTs of both HSA 18 and HSA 19 stay close to the nuclear center. Let us further assume that HSA 18 CTs are strongly attached to the lamina, while HSA 19 CTs are not. Accordingly, both of the HSA 18 CTs but not the HSA 19 CTs should follow the nuclear envelope during shape transformation. At the end of the transformation from a flat-ellipsoidal to a spherical nucleus, we would expect the two HSA 18 CTs to now be located in the nuclear periphery far away from the 3D nuclear center. Both HSA 18 CTs may be still close together or located at opposite sites of the now spherical nucleus. Accordingly, we would expect strongly bimodal angle distributions for the HSA 18 CTs, with one peak at small angles and a second peak at large angles. When we recorded the angular separation of the two peripherally located HSA 18 CTs in a set of 82 lymphocyte nuclei from peripheral human blood, we found indeed two maxima, one around 10° and a second at 140° (J. Hase and M. Cremer, unpublished data).

Let us now consider the opposite transformation of a spherical precursor nucleus, with both HSA 18 CTs located peripherally either close to each other or on opposite sides, to a flat-ellipsoidal nucleus. There are an infinite number of possible mid-planes through the center of a spherical nucleus. In order to achieve the desired location of both CTs close to the 3D center, it is essential to choose the correct mid-plane for this transformation. Consider a population of spherical nuclei with two HSA 18 CTs located at opposite sides. We need to choose mid-planes roughly perpendicular to the connection between the IGCs of the two CTs. Selection of a mid-plane along this connection would yield a transformation with both CTs located at the nuclear rim.

The considerations above emphasize that nuclear shape transformations may affect CT arrangements [47], and inspire a number of questions for future experiments. Do shape changes enforce changes of CT arrangements or vice versa? And are changes of nuclear shape causally connected with changes of gene expression patterns?

### Mechanisms Responsible for the Establishment of Cell-Type-Specific CT Arrangements

We expect that complex genetic and epigenetic mechanisms at various hierarchical levels act in concert in order to establish, maintain, or alter higher-order chromatin arrangements as required for proper nuclear functions. A search for such mechanisms must take into account the fact that chromosome arrangements—at least in the cell types studied so far—follow probabilistic and not deterministic rules [42]. Candidate mechanisms include the genetically regulated timing of centromere separation and anaphase movements of individual chromosomes [48], interactions of constitutive and facultative heterochromatic domains with each other and the nuclear lamina, and interactions of chromatin domains comprising active genes with nuclear bodies located in the interchromatin compartment [4].

The mechanisms producing a size-correlated radial positioning in fibroblast nuclei during telophase and early G1 may differ from the mechanisms acting to establish this arrangement in mitotic rosettes. The central part of a mitotic spindle in vertebrates consists of a tight bundle of microtubules stretched between the two centrioles. The exclusion of chromosomes from this tight bundle leads to the formation of a centromere ring around this bundle. Accordingly, the localization of the IGCs of smaller chromosomes towards the center of the rosette may simply reflect their small size. Geometrical constraints may force large chromosomes to expand their arms to the periphery of the rosette, and accordingly, their IGCs are shifted towards the periphery. To some extent the spindle may reinforce a size-correlated radial arrangement of chromosomes (see [12] and references therein). Persistence of this order from the prometaphase rosette to the anaphase rosette could yield a starting configuration for the decondensation of chromosomes in telophase and early G1 consistent with size-correlated radial CT arrangements in the flat-ellipsoidal fibroblast nuclei. If cycling lymphoblasts were to show the same size-correlated radial chromosome arrangement in mitotic rosettes—a possibility not yet experimentally tested—the gene-density-correlated radial arrangements during interphase would require major chromosome movements during telophase/early G1.

### Evolutionary Aspects

Systematic comparisons of nuclear architecture in cell types from evolutionarily distant species must be performed to distinguish evolutionarily conserved features from species-specific ones. One feature of nuclear architecture has already been detected that has been strongly conserved during evolution. In animal species, ranging from *Hydra* to mammals [37,49,50], and plant species [51], DNA replication is linked to a typical nuclear topology of early-, mid-, and late-replicating chromatin. The evolutionary conservation of a layer of constitutive and facultative heterochromatin at the nuclear periphery argues for a selective advantage.

In 1975, T. C. Hsu proposed the “bodyguard” hypothesis for the function of constitutive heterochromatin [52]. Hsu argued that heterochromatin localized at the nuclear periphery might protect the centrally localized euchromatin against mutagens, clastogens, and viruses entering the nucleus. If this hypothesis is correct, we should observe DNA damage preferentially in the peripheral chromatin shield. More recently, a protective, “buffering” role for redundant DNA was suggested [53]. However, experimental evidence to support these hypotheses has not been provided so far.

Finally, we may consider the possibility that a layer of dense facultative and constitutive heterochromatin intimately connected to the nuclear lamina may provide more stability to the nucleus, just as certain proteins beneath the cell membrane provide stability against mechanical stress. This protection may be particularly important in cell types, such as muscle cells, that are the frequent subject of mechanical stress, affecting the shape of both the cell and its nucleus [54,55].

## Materials and Methods

### 

#### Cells, fixation procedure, and 3D FISH pretreatment

A vigorously growing primary human fibroblast culture was established from a skin biopsy of a 2-y-old boy. Chromosome banding and M-FISH analyses performed after the second passage (1:2 split) showed a normal male karyotype (46, XY). Surplus cultures were kindly provided by the Abteilung Medizinische Genetik, Munich University, Germany; cells were further grown in our laboratory in DMEM medium supplemented with 10% FCS, and aliquots were frozen at about 5–7 passages in liquid nitrogen. Cells from these aliquots were further propagated for 2–4 wk, subcultivated (1:2) every 5 d, and routinely checked for absence of mycoplasm contaminations [56]. For 3D FISH and ReFISH experiments, cells were seeded on coverslips (26 × 76 mm, thickness 0.17 ± 0.01 mm). For studies of quiescent cell populations, cultures were grown to confluence and maintained for 1 wk before 3D fixation was performed in 4% paraformaldehyde/1× PBS for 10 min [30]. Control experiments with BrdU pulse labeling (1 h) and immunostaining of the cell-cycle-specific nuclear protein Ki67 indicated that more than 99.5% of the cells were in a quiescent state (G0) under these conditions. To investigate G0 and S-phase cells simultaneously on the same coverslip, cells were fixed at approximately 40%–50% confluence. Nuclei from cells in and out the cell cycle were discriminated by BrdU pulse labeling (45 min) and pKi67 staining. Growing fibroblast cultures were also used to obtain prometaphase rosettes. Nuclei in S-phase showed pKi67 staining and incorporation of BrdU, while G0 nuclei lacked both signals (data not shown). An amniotic fluid cell culture from a female fetus (46, XX) was established following diagnostic amniocentesis, and a growing early-passage culture was pulse-labeled with BrdU (45 min) prior to 3D fixation. Permeabilization steps performed prior to 3D FISH included treatments with 0.5% Triton-X100 (20 min), 20% glycerol in PBS (30 min), repeated freeze/thawing in liquid nitrogen, and incubation in 0.1 M HCl (5 min) or pepsin (0.002% in 0.01 M HCl). Slides were stored at 4 °C in 50% formamide/2× SSC until 3D FISH was performed.

#### DNA probes, labeling protocols, 3D FISH, and probe detection

Whole chromosome painting probes were kindly provided by Malcolm Ferguson-Smith (Cambridge University, United Kingdom). Probes were established from flow-sorted human chromosomes and amplified by DOP-PCR.

For 24-color 3D FISH experiments in a single assay, chromosome paint probes for all 24 chromosome types (HSAs 1–22, X, and Y) were labeled using a combinatorial labeling scheme with seven differentially labeled nucleotides including diethylaminocoumarine (DEAC; NEN Life Science Products, Zaventem, Belgium), Fluorogreen (Amersham Pharmacia Biotech, Piscataway, New Jersey, United States), TexasRed (Molecular Probes, Eugene, Oregon, United States), Cy3, Cy5 (Amersham Pharmacia Biotech), biotin-dUTP (Rockland Immunochemicals, Gilbertsville, Pennsylvania, United States) and digoxigenin-dUTP (Roche, Basel, Switzerland) [31]. A mix containing the 24 labeled probes in 50% formamide/10% dextran sulfate/1× SSC was hybridized for 3 d at 37 °C as previously described [30]. Post-hybridization washes were performed three times with 0.5× SSC at 60 °C. Avidin-Cy5.5 was used for detection of biotin and anti-dig-Cy7 (custom-made) for the immunodetection of digoxigenin.

For the differential coloring of all 24 chromosome types in ReFISH experiments [30,32], chromosome-specific paint probes from all 24 human chromosome types were labeled with either biotin-dUTP, TAMRA-dUTP, or Spectrum Green–dUTP. Two different hybridization mixtures were prepared in a way that allowed the unequivocal discrimination of each chromosome type in two subsequent hybridization experiments. After hybridization of the first probe subset, biotinylated probes were detected by Avidin-Cy5.5. Confocal image stacks were acquired as described below, and the cell coordinates were recorded. Thereafter, the same cells were re-hybridized with the second probe subset, followed again by detection with Avidin-Cy5.5 and confocal microscopy.

In two-color 3D FISH experiments aimed at the simultaneous visualization of two pairs of homologous CTs, we used biotin- and digoxigenin-labeled chromosome paint probes for HSAs 18/19, HSAs 17/Y, and HSAs 1/20.

#### Microscopy

After 3D FISH of all 24 chromosome types in a single assay, nuclei were imaged with an epifluorescence wide-field microscope (DMRXA, Leica, Wetzlar, Germany), equipped with a Plan Apo 63×/1.4 oil immersion objective, an 8-filter wheel with narrow-band filters (Chroma Group, San Bruno, California, United States), and an automated z-step motor [57]. For image capturing, a Sensys CCD camera (PhotoMetrics, Huntington Beach, California, United States) was used. Both camera and microscope were controlled by Leica QFluoro software. Stacks of optical sections with an axial distance of 250 nm were collected from nuclei with a regular shape showing apparently complete and specific hybridization signals in all channels. For each optical section images were collected sequentially for all fluorochromes. Stacks of 8-bit gray-scale 2D images were obtained with a pixel size of 110 nm in the *x* and *y* directions, and an image size of 256 × 256 pixels.

In ReFISH experiments, images for the three fluorochromes were obtained from the same nuclei after the first and second hybridization with a Zeiss (Oberkochen, Germany) LSM 410 confocal microscope equipped with a 63× Plan Apo objective and filters for FITC, Cy3, and Cy5. Scans were sequentially performed for the three fluorochromes on each light-optical section. An alignment of the two image stacks obtained for each nucleus after the first and second hybridization was performed on a Silicon Graphics (Mountain View, California, United States) workstation (OS Irix 6.2) using the program Correlator [58] in the integrated development environment Khoros (Khoral, Albuquerque, New Mexico, United States). This procedure allowed the fitting of the two image stacks with subvoxel accuracy. A comparison of the nuclear shape after the first and second hybridization did not reveal a notable difference, but we observed a slight increase in volume, which was corrected by the computer algorithm.

#### Deconvolution and image processing

Each fluorochrome channel was normalized to a maximum intensity value of 255, and subjected to deconvolution by the software Huygens (Scientific Volume Imaging, Hilversum, The Netherlands). Thereafter, chromosomes in the image stacks were classified according to their labeling scheme using the software goldFISH [33] running on a Silicon Graphics workstation. This software carried out an automated classification of fluorescently stained areas in each light-optical nuclear section on the basis of the combinatorial labeling scheme. A false color representing the classified chromosome type was allocated to each classified territory. The software calculated the 3D IGCs of each classified territory as well as the CN or the CR by means of the DAPI image stack. Maximum intensity projections of image stacks were made with ImageJ software (National Institutes of Health, Bethesda, Maryland, United States). Displayed overlays were processed with Adobe Photoshop (Adobe Systems, San Jose, California, United States). Three-dimensional reconstructions of image stacks were performed using Amira 2.3 (Mercury Computer Systems, Chelmsford, Massachusetts, United States). Overlap of CTs in nuclei or of chromosomes in 3D fixed mitotic rosettes can be a source of misclassification. Because of the remarkable flatness of fibroblast nuclei in G0 (maximum height approximately 6 μm, with CTs often expanded from the bottom of the nucleus to the top), errors due to CT overlaps are less likely than in spherical nuclei.

#### Data evaluation

For each classified PC and CT we determined its 3D IGC, together with the CR and the CN, respectively. IGC coordinates were imported into Excel (Microsoft, Redmond, Washington, United States), and the following distances and angles were measured: (1) 3D CR–PC and 3D CN–CT distances (3D radial distances), (2) 3D PC–PC and 3D CT–CT distances between all possible pairs of homologous and heterologous PCs or CTs, and (3) 3D PC–CR–PC and 3D CT–CN–CT angles. For comparison of different nuclei, 3D distances were normalized using the following procedure. A coordinate system was applied to each individual nucleus with the nuclear center as the origin (polar coordinates). The angle α between the longer cell axis and the *x*-axis of the coordinate system was determined, and the coordinates were recalculated following a rotation: *x′* = cosα *x* − sinα *y* and *y′* = sinα *x −* cosα *y*. For size measurement of nuclei, the DNA was stained with DAPI or TOPRO-3. The diameters in *X* and *Y* were then measured using the light-optical section with maximum lateral nuclear expansion. The height was measured between the upper and lower plane showing the most peripheral DAPI staining along the *z*-axis. The relative radial distance *r* of a PC or CT was calculated as *r* = (*r*_1_/*r*_0_)·100, where *r*_1_ represents the distance CN–CT or CR–PC, respectively, and *r*_0_ denotes either the distance between the CR and the prometaphase edge or between the CN and the nuclear edge, drawing a line through the IGC of the analyzed chromosome. Angles were calculated between the IGCs of homologous CTs or PCs using the CN or CR as the midpoint. The relative radial distance between heterologous PCs or CTs was calculated as a fraction of the nuclear diameter.

Distance and angle measurements obtained for IGCs of CTs and PCs were compared with distances and angles calculated between points statistically placed by a random number generator (“random point distribution model”).

MDS plots were generated by SPSS 11 (SPSS, Chicago, Illinois, United States). This program provided a 2D distance map taking into account the normalized mean heterologous 3D CT–CT distances calculated for all possible combinations of heterologous CTs. All distances were normalized to the diameter of the nucleus before generating the plots. The units after the transformation to a MDS map are arbitrary since the distances are merely relative. For a quantitative 3D evaluation of CT distributions in two-color 3D FISH experiments, the 3D-RRD computer program was used (see [25] and [59] for detailed description). Briefly, the program determines (1) the center of gravity of a given nucleus and its borders, on the basis of the DNA counterstain, and (2) all voxels of painted chromosome territories. The nuclear radius in any direction from the nuclear center of gravity to the segmented nuclear edge was normalized to 100%, and the nuclear space was divided into 25 concentric shells. All shells possessed the same thickness along each possible radial vector from the center of the nucleus to the periphery. Accordingly, the thickness of these shells in flat ellipsoidal nuclei was much larger along the *x*- and *y*-axis than along the *z*-axis. Thus, the distribution of the DNA of painted CTs was measured and expressed as a function of the relative distances of each shell from the center of the nucleus. Significance tests were carried out with either SPSS 11 or Sigma Stat (SPSS). If not otherwise stated a K-S significance test was applied.

#### Modeling of human fibroblast cell nuclei with statistical CT distributions

To simulate the statistical distribution of CTs in human fibroblast nuclei, the SCD model was applied [25,41,60]. The DNA content of individual human chromosomes [61] was used to estimate the number of 1-Mbp chromatin domains constituting a given model chromosome. These domains were represented by 500-nm diameter spheres. Model nuclei were generated with an ellipsoidal shape with half-axes representing the average half-axes of human G0 fibroblast nuclei determined from light-optical stacks (*x* = 10 μm, *y* = 5 μm, and *z* = 2.5 μm).

Briefly, starting configurations representing a statistical chromatid distribution in male diploid human fibroblasts (46, XY) at late anaphase/telophase were established as follows. The location of the center of gravity of each chromatid was initially represented by the mass center of a small inelastic sphere (iS). The volume of a given iS was proportional to the DNA content of its natural chromosome counterpart, while its radius was very small compared with the half-axes of the ellipsoid. The total volume of the 46 iSs (representing the 46 chromosomes of the diploid human complement) comprised 22% of the total volume of the ellipsoidal model nucleus. The mass centers of the 46 iSs were statistically placed into the ellipsoid as follows. Using a random-number generator, the 3D coordinates for the mass centers of all iSs were generated iteratively in a nonoverlapping fashion. If the addition of a new iS yielded any overlap with the position of already existing iS, the 3D coordinates were discarded and new randomly generated 3D coordinates were tested. This process was repeated until all 46 iSs were located in the model nucleus. In a second step, chromatids were modeled as small rods with a spherical cross section of 500 nm (see Figure 1D). Each rod represents a linear chain of spherical 1-Mbp chromatin domains. To model the dense packaging of 1-Mbp domains in chromatids, a distance of 13 nm was simulated between the centers of gravity of any two adjacent 1-Mbp domains along the chromatid axis. For example, a rod representing a chromatid with a DNA content of 100 Mbp had a length of 1,300 nm. In this way we modeled highly compacted and rigid chromatids. Rods were placed with a random orientation inside the 46 iSs in such a way that their centers of gravity coincided with the centers of the iSs from step one. As a third step, the relaxation of the statistically placed chromatids into decondensed CTs was simulated. For this purpose Monte Carlo relaxation loops were carried out with about 400,000 steps to obtain thermodynamic equilibrium configurations [14]. For decondensed CTs we assumed 120-kb linker connections between neighboring 1-Mbp chromatin domains, representing higher-order clusters of 100-kb loop domains [62]. These connections were modeled by entropic spring potentials enforcing a mean distance of 600 nm between the centers of gravity of adjacent domains. For distances between the centers of two adjacent 1-Mbp domains of 500 nm or more, we assumed that their repulsive potential was zero. When distances became smaller than 500 nm, the repulsive potential became increasingly positive, resulting in an increasing mutual repulsion between the two domains. Long-term Monte Carlo relaxation loops showed that the two assumptions of a spring potential and a repulsive potential are not sufficient to maintain the experimentally observed compactness of CTs. To achieve model CTs with diameters comparable to CTs in fibroblast nuclei, we introduced a weak potential barrier around each simulated chromatin domain chain representing a given chromosome. In each Monte Carlo step the 3D coordinates of a randomly chosen 1-Mbp domain were changed slightly for each CT. The new coordinates were accepted if the resulting chromatin domain configuration came closer to the thermodynamic equilibrium. Otherwise they were rejected and the process repeated. The 3D positions of the CT centers of gravity after 400,000 Monte Carlo steps represented the statistical arrangement of CTs in model nuclei. The further evaluation of distances and angles between model CTs was performed as described for experimental fibroblast nuclei.

## Supporting Information

Figure S1 Male Human Fibroblast Nucleus (G0): Deconvolution 3D Microscopy, CT Classification, and 3D Reconstruction Images selected from a stack of 20 light-optical serial sections. Sections from a fibroblast nucleus following chromosome painting with 24-color M-FISH were recorded by wide-field epifluorescence microscopy. Images from left to right show optical sections obtained from the bottom to the top of the nucleus.
(A) RGB images without deconvolution.
(B) RGB images after deconvolution.
(C) False color images after classification.
(D) Still shot from Video S1, which shows the simulation of CT expansion in a fibroblast model nucleus according to the SCD model (compare with Figure 1).
(766 KB JPG).

Figure S2 Positions of IGCs of Each CT in G0 Human Fibroblast Nuclei Each point represents the 2D projection of the ICG of an individual CT. The *x*- and *y*-half-axes of the ellipses shown represent the mean *x*- and *y*-half-axes of all the 54 nuclei evaluated. Each nucleus was rotated until its long axis fitted the abscissa of the coordinate system. The inner ellipsoid lines were drawn to facilitate the comparison of the ICG locations from different CTs.
(1.5 MB JPG).

Figure S3 Prometaphase Rosettes: Positions of IGCs of Each Chromosome 2D projections of the IGCs determined for all chromosome types (1–22, X, and Y) present in 28 prometaphase rosettes of male diploid human fibroblasts (compare to Figure S2).
(1.2 MB JPG).

Figure S4 Cumulative Normalized 3D Radial Distances in Human Fibroblast Nuclei (G0) Cumulative frequency of normalized 3D CN–CT distances for all chromosome types (1–22, X, and Y) measured in 54 G0 human fibroblast nuclei of the 2D projections shown in Figure S2. The origin on the abscissa corresponds to the CN, and “1” corresponds to the maximum 3D distance from CN to any site of the nuclear periphery. Distances were sorted into ten classes representing ten concentric 3D nuclear shells. To define these shells, normalized 3D radii were subdivided into ten equal segments.
(919 KB JPG).

Figure S5 Prometaphase Rosette: Deconvolution 3D Microscopy, PC Classification, and 3D Reconstruction Prometaphase rosette from a male fibroblast following chromosome painting with the 24-color M-FISH protocol.
(A–C) Typical sections selected from a stack of 20 light-optical serial sections obtained by wide-field epifluorescence microscopy (A) before deconvolution, (B) after deconvolution, and (C) after classification.
(D) 3D reconstruction from the entire stack of deconvoluted light-optical images.
(E) Mid-plane section recorded in eight channels. Note that the Cy7 channel was not analyzable in this experiment. As a consequence, Chromosomes 5 and 19 were both labeled solely by Spectrum Green, while Chromosomes 8 and 20 were labeled solely by Cy3. Despite these limitations, differences in size and labeling intensity allowed the classification of these PCs.
(876 KB JPG).

Figure S6 Cumulative Normalized 3D Radial Distances in Human Fibroblast Prometaphase Rosettes Cumulative frequency plot of normalized 3D CR–PC distances for all chromosome types (1–22, X, and Y) measured in 28 rosettes of the 2D projections shown in Figure S3. The origin on the abscissa corresponds to the CR, and “1” corresponds to the maximum 3D distance from CR to any site of the rosette periphery. Distances were sorted into ten classes as in Figure S4.
(922 KB JPG).

Figure S7 Mean Normalized 3D CN–CT/CR–PC Distances and Standard Deviation of All Chromosome Types in Human Male Fibroblasts Left: normalized mean radial 3D CN–CT distances from the 54 evaluated 3D fibroblast nuclei. Right: normalized mean radial 3D CR–PC distances of the 28 evaluated 3D prometaphase rosettes. SD, standard deviation.
(424 KB JPG).

Figure S8 Comparison of Radial CT Positions in Quiescent and Proliferating Fibroblasts (A–C) Left: for quality control chromosome paint probes were hybridized to metaphase spreads from phytohemagglutinin-stimulated human lymphocytes. For example, shown are metaphase spreads after two-color 3D FISH with the following pairs of chromosome paint probes: (A) HSA 18 (red) and HSA 19 (green); (B) HSA 17 (green) and HSA Y (red); and (C) HSA 1 (red) and HSA 20 (green). Painted metaphase chromosomes show a rather homogeneous coverage, except for the centromere region, which remained unstained because of the signal suppression with C_o_t-1 DNA. Right: maximum intensity projections of confocal image stacks from selected nuclei in quiescent (G0) and proliferating (early S-phase) human male fibroblasts demonstrate the variability of CT positioning. CT colors are the same as described for metaphase chromosomes.
(D) To compare the voxel-based analysis of 3D radial CT 18 and CT 19 distributions in G0 and S-phase fibroblasts (see Figure 6A and 6D) with an IGC-based 3D analysis, we allocated CT 18 IGCs and CT 19 IGCs to one of five concentric nuclear shells with equal volume. Shell 1 corresponds to the most peripheral shell. The data was analyzed in this way to compare with Figure 1 in Bridger et al [27], except that 3D distances were used here. In both quiescent and S-phase nuclei a higher fraction of CT 19 IGCs was found in the two central shells 4 and 5 than for CT 18 IGCs. In contrast, in S-phase nuclei the fraction of CT 18 IGCs was larger in the more peripheral shells 1–3. In quiescent nuclei only shells 2 and 3 revealed a higher fraction of IGCs from HSA 18 CTs, while the fraction of IGCs from HSA 19 CTs was slightly higher in the most peripheral shell, shell 1. The results are consistent with a voxel-based approach (see Figure 6).
(E) Comparison of the median and the mean 3D CN–CT distances obtained for HSA 18 CTs and HSA 19 CTs in G0 and S-phase nuclei. A plus sign indicates a slight shift of CT 18 IGCs towards the nuclear periphery in early S-phase fibroblast nuclei compared to G0 nuclei, whereas a minus sign indicates a shift to the interior of the CT 19 IGCs. However the measured shifts were statistically not significant (ns).
(F) Median and mean normalized 3D CN–CT 18 distances were not significantly different from CN–CT 19 distances in G0 fibroblast nuclei, but showed a marginal difference (* *p* < 0.05) in early S-phase nuclei.
(1.3 MB JPG).

Figure S9 G0 Nuclei: Mean Angular Separation between 3D IGCs of Homologous CTs and the CN CT–CN–CT angles were measured between the IGCs of homologous CTs and the center of the nucleus (CN) in 54 G0 fibroblast nuclei. Sample sizes *(n)* indicate the number of nuclei in which CT–CN–CT angles could be measured for a given pair of homologous CTs. The experimental distribution did not deviate from a normal distribution (*p >* 0.05; one-tailed K-S test of goodness of fit). With few exceptions pairwise comparisons of the mean angular separation between a pair of homologous CTs with the respective mean angle distribution in 60 random point distribution model nuclei did not show a significant difference (*p* > 0.05; two-tailed K-S test). Significant differences (*p* < 0.05) are indicated by an asterisk. The comparison of mean angular separation of homologous CTs with statistically placed homologous CTs in 50 SCD model nuclei also did not reveal significant differences except for 4-CN–4 and 20-CN–20 angles, which were smaller in the experiment than in the model (**p < 0.01).
(503 KB JPG).

Figure S10 Prometaphase Rosettes: Mean Angular Separation between 3D IGCs of Homologs and the CR PC–CR–PC angles between the IGCs of homologous PCs and the rosette center (CR) measured in 28 prometaphase rosettes. No significant difference was detected in comparison with angular separations found in the random point distribution model (*p* > 0.05; two-tailed K-S test).
(426 KB JPG).

Figure S11 Significance Levels for Pairwise Comparisons between Heterologous 3D CT–CN–CT Angles in 54 G0 Fibroblast Nuclei Significance levels were determined by the two-tailed K-S test. Green, not significant, *p* > 0.05; yellow, *p <* 0.05; red, *p <* 0.01. Minus/plus signs in a colored field indicate that the chromosome pair given on the left shows a significantly shorter/greater mean radial distance than the chromosome pair presented at the top.
(A) Large chromosomes: HSAs 1–5.
(B) Small, acrocentric chromosomes: HSAs 13, 14, 15, 21, and 22.
(C) Other small chromosomes: HSAs 16–20.
(292 KB JPG).

Figure S12 Arrangements of HSA 7 and HSA 8 CTs in 50 Fibroblast Nuclei Nuclei of G0 fibroblasts were subjected to 3D FISH with painting probes for HSA 7 and HSA 8, labeled with dUTP-Cy3 (green) and dUTP-FITC (red), respectively. Maximum intensity projections of confocal image stacks from 50 scanned nuclei are shown to demonstrate the variability of proximity patterns (for quantitative measurements see Figure S13).
(870 KB JPG).

Figure S13 Quantitative Evaluation of the Angular Separation of Homologous and Heterologous Pairs of HSA 7 and HSA 8 CTs in 50 Fibroblast Nuclei Upper left: Ellipses represent the normalized 2D shape of the nuclei and show 2D projections of the radial IGC locations of HSA 7 and HSA 8 CTs. Upper right: The IGCs of CT 7 and CT 8 pairs, respectively, were rotated around the nuclear center until one IGC lay on the positive abscissa (closed circles). Open circles show the IGC position of the corresponding homolog. Below are the mean 3D CT–CN–CT angles between homologous and heterologous HSA 7 and HSA 8 CTs, their ranges, and their standard deviations. Angles between homologous and heterologous pairs showed a normal distribution. Comparisons of experimental data with mean 3D angle distributions in the random point distribution model or SCD model did not show a significant difference (*p* > 0.05; two-tailed K-S test).
(328 KB JPG).

Video S1 Model Nucleus: CT Simulation The video shows the simulation of CT expansion in a fibroblast model nucleus according to the SCD model (compare with Figure 1).
(567 KB MPG).

## Abbreviations

[number]D: [number]-dimensional
CN: intensity gravity center of the nucleus
CR: intensity gravity center of the prometaphase rosette
CT: chromosome territory
FCS: fetal calf serum
FISH: fluorescence in situ hybridization
HSA: Homo sapiens chromosome
IGC: intensity gravity center
iS: inelastic sphere
K-S: Kolmogorov-Smirnov
MDS: multidimensional scaling
M-FISH: multiplex fluorescence in situ hybridization
NOR: nucleolar organizer region
PC: prometaphase chromosome
SCD: spherical 1-Mbp chromatin domain
*t*-test: Students *t*-test
*U*-test: Mann-Whitney *U*-test
